# Ten simple rules for queer data collection and analysis by STEM researchers

**DOI:** 10.1371/journal.pcbi.1013091

**Published:** 2025-05-28

**Authors:** Dori M. Grijseels, M. Banqueri, Keerthana Iyer, Lee Hanlin, Melanie Ortiz Alvarez de la Campa, David Pagliaccio, Bittu K. Rajaraman, Eitan Schechtman, On behalf of ALBA Network Gender & Sexual Diversity Working Group

**Affiliations:** 1 Social Systems and Circuits Group, Max Planck Institute for Brain Research, Frankfurt am Main, Germany; 2 Centre for Discovery Brain Sciences, University of Edinburgh, Edinburgh, United Kingdom; 3 ALBA Network, Federation of European Neuroscience Societies (FENS), Brussels, Belgium; 4 Department of Neurobiology, University of California San Diego, San Diego, California, United States of America; 5 Department of Molecular Microbiology and Immunology, Brown University, Providence, Rhode Island, United States of America; 6 Department of Psychiatry, Columbia University, New York, New York, United States of America; 7 Division of Child and Adolescent Psychiatry, New York State Psychiatric Institute, New York, New York, United States of America; 8 Department of Psychology, Ashoka University, Sonipat, Haryana, India; 9 Department of Biology, Trivedi School of Biosciences, Ashoka University, Sonipat, Haryana, India; 10 Department of Neurobiology and Behavior and Center for the Neurobiology of Learning and Memory, University of California, Irvine, California, United States of America; Carnegie Mellon University, UNITED STATES OF AMERICA

## Abstract

Queer people are still underrepresented both as STEM researchers and participants, partially due to a dearth of accurate data on this demographic. The lack of consideration for queer identities in data collection and dissemination causes a vicious cycle of exclusion. To address this invisibility, it is important to collect and report data in an inclusive and accurate manner across all areas of research, including in studies that are not specifically focused on queer populations. However, STEM researchers are often unsure of how to properly collect data in a manner that fairly represents queer people. We have developed a list of Ten Simple rules to aid researchers to perform data collection on queer individuals, focusing on study design and data dissemination. We address several issues in queer data, such as language use, dealing with small populations, and balancing demands. We also discuss how to extend this inclusive practice for studies on animal populations. These rules are aimed at anybody surveying populations which may contain queer individuals, including for example research studies and inclusivity surveys for conferences. By providing practical tips, we hope to alleviate insecurity and confusion around this topic.

## Introduction

Queer, or LGBTQIA+ (Lesbian, Gay, Bisexual, Transgender, Queer, Intersex, Asexual), individuals continue to face barriers in STEM (science, technology, engineering, and math) that their straight and cisgender counterparts do not [1–6]. These barriers are part of a vicious cycle of exclusion and low retention rates of queer individuals within STEM, leading to under-representation among STEM researchers [1,5,7], under-funding of studies into queer experiences in STEM, and the persistence of historic and systemic barriers for queer people. This, in turn, results in a lack of consideration for queer identities among research participants in STEM [8–10], for example in the domains of psychology and biology. Furthermore, adopting a simplified binary approach to sex in biological and medical studies obscures complex mechanisms, through which factors such as genes, hormones, and behavior determine sex diversity [11,12]. Although it has become commonplace to include binary sex (i.e., male/female) or gender (i.e., man/woman) across studies, non-binary identities are often not considered [13] (for more discussion on this, see rule 1). In addition, transgender status and sexual identity are often omitted, and if they are included, only a limited number of labels are available [14,15]. Consequently, the demographics included in such studies are wholly insufficient to capture the queer experience, and its effect on any dependent variable that is being measured. This results in data from queer people not being fully evaluated, which perpetuates a vicious cycle of systemic exclusion of these individuals.

To combat the invisibility, both for queer individuals who are part of experimental cohorts, and STEM researchers who identify as queer, it is important to collect data reflecting gender and sexual diversity [5]. Recent appeals to collect such data, both within STEM fields [16] and beyond [17], have focused on multiple domains, including national surveys [18,19], professional organizations [20], and individual conferences [21]. As such, sex, gender, and sexual orientation are increasingly included as key demographics across surveys—and omission of these variables has recently been rightfully criticized [18]. However, the collection of data on queer individuals and design of surveys or questionnaires to do so may be challenging for STEM researchers with little or no formal training in collecting data of this type, and who are unfamiliar with queer identities.

One obstacle to collecting and analyzing data on queer identities is the complexity and diversity of this population. The queer identity itself consists of multiple axes, including gender identity and sexual orientation. Each of those axes contains multitudes, for example sexual orientation may consist of a combination of numerous experiences, including romantic attraction, sexual attraction, and gender identity [22]. In non-human settings, such as in animal studies, this diversity mainly derives from the multitude of systems involved in determination of sex and sexual behavior, such as genes, hormones, and behavioral context [11,12]. As such, collecting and analyzing data on sex, gender, and sexual orientation is not trivial, and it is important that anyone collecting such data critically considers the study design, as well as the data analysis plan following data collection [23].

In addition, queer identities are often both politicized [6] and misunderstood, leading individuals who are unfamiliar with this community to be uncertain about what terms should and should not be used. A prime example is the word ‘queer’, which was historically seen as a slur, but has become increasingly popular as a term both for the community as a whole, and for individuals as an expansive label to describe their own gender and/or sexual identity [15,23]. Terms may also vary across nations, languages, and cultural groups; and experiences of queer identity, especially in the case of transgender and gender diverse (TGD) individuals, may depend on cultural context [24].

**Fig 1:**
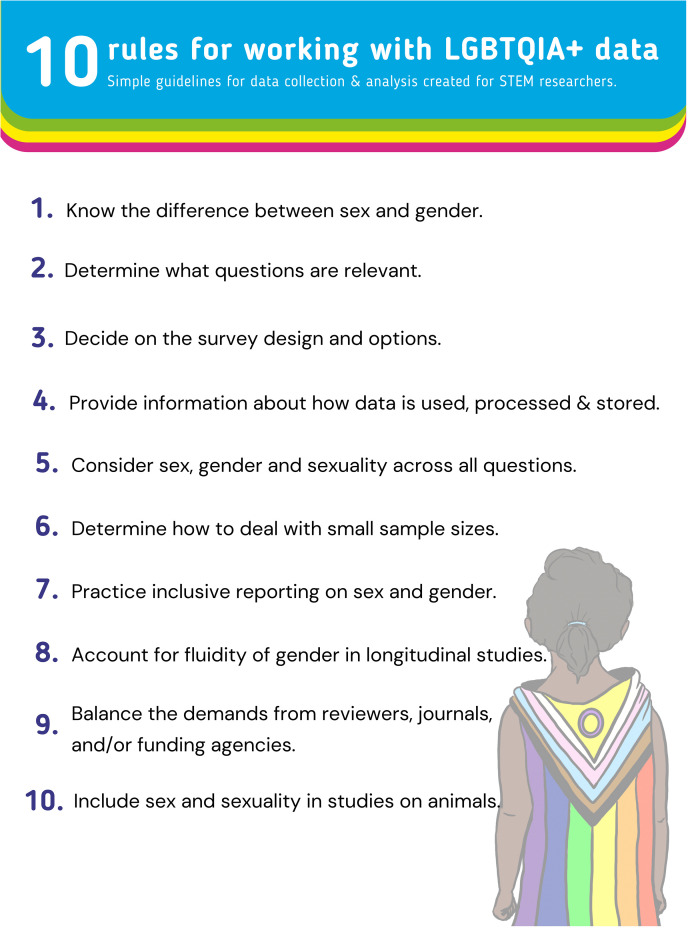
Ten rules for working with LGBTQIA **+** **data.** Graphical depiction of the ten simple rules described in this manuscript.

In order to reconcile the wonderfully complex, but understudied, queer experience on the one hand, and the limited resources STEM researchers often face when collecting data on queer individuals, we adapted a set of guidelines originally developed with the ALBA network, a network promoting inclusion in the brain sciences. These guidelines were originally written to guide brain scientists in more inclusive data collection practices (S1 and S2 Files) [23]. Here, we condensed these guidelines into ten simple rules for researchers across all STEM disciplines to consider when collecting and analyzing data which includes information on sex, gender or sexual diversity (Fig 1). Building on previously published recommendations for data collection and analysis [7,13,17,10,25–30], we provide actionable tips specifically tailored to STEM researchers across all fields. These guidelines apply to a wide variety of situations where demographic data may be relevant, ranging from psychological and biological experiments, to demographic surveys for inclusion purposes. These ‘rules’ are best served as guidelines, since queer identities, and the language and norms surrounding them, are continually changing and vary from culture to culture. As such it is important to seek advice from local interest groups and diversity advocates within the local community, and continue engaging with discussions on queer inclusion in STEM.

### 1. Know the differences between sex and gender

Sex and gender are often conflated as a single, binary concept [13,26]. However, these two concepts are not synonyms, nor are either of them binary. Sex may be interpreted in a number of different ways. An often used interpretation is the sex assigned at birth, i.e., the sex that was originally noted on the birth certificate. Another definition of sex is the legal sex, e.g., the sex that is listed on the passport. What options are available for either of these definitions, depends on the country. Although for most people the sex assigned at birth and the legal sex will overlap, this may not always be the case. Sex is often an irrelevant variable outside of biological or medical settings, while gender is more likely to influence how a person is treated and affected by their environment, and often a more accurate representation of a person’s experiences and sense of self. For example, when it comes to discrimination, this is usually based on the perceived gender, and the extent to which a person confirms to this gender, not the legal sex or sex assigned at birth. A famous example is the case of Ben Barres, an acclaimed neuroscientist and trans man. After giving a seminar, an attendee remarked “Ben Barres gave a great seminar today, but then his work is much better than his sister’s.” [31] Ben Barres did not have a sister, and the attendee was referring to his own work under his previous name.

If there is a reason to ask for sex, it should be made clear what definition of sex is used, and why the question is asked, so that participants can answer it as accurately as possible. In all questions about one’s sex, the option for intersex should be included. Intersex, sometimes referred to as DSD (difference in sex development), refers to people who were born with a variation of physical sex characteristics. Although there has been a recent increase in studies on their experiences, they are often still excluded [29]. Because intersex people are often assigned a binary (male/female) sex at birth and a binary legal sex, it is best to include a separate question specifically asking for intersex status [32]. Furthermore, in some countries (e.g., Germany, India and Australia) intersex people may have the option of a third sex marker on their passport and/or birth certificate, thus including this is as an option in any questions about sex is advised. It should particularly be noted that intersex does not equate to a non-binary gender; intersex people can identify as any gender, regardless of their sex, in the same way endosex (i.e., people who are not intersex) people can.

Gender is a complicated and multi-faceted concept, and people’s self-identified gender may be nuanced and complex [13]. For TGD people, their gender is usually different from their sex assigned at birth. They may have a binary gender (i.e., man or woman), another gender outside of the binary (non-binary is an umbrella term used for these genders), no gender (agender), or a gender which changes (e.g., genderfluid). Even this list is not exhaustive of the way people experience their gender. Gender is self-identified, may change during one’s lifetime, and often comprises a number of different aspects such as individual expression and societal roles.

### 2. Determine what questions are relevant

Depending on the goal of the data collection you may want to collect data on a person’s gender, sex, sexuality, and other personal characteristics. However, it is important to carefully consider what is actually relevant [13,30], as well as what you are legally allowed to ask. For example, if you are organizing a conference, you may be interested in your participants’ gender to determine statistics for equality measurements. In this case, you could simply ask participants about their gender identity without including questions about sex, which will be irrelevant (as discussed in rule 1). Conversely, if you’re performing a study on uterine health, gender will be a poor approximation, and it is advised to specifically ask if your participant has a uterus instead. Note that some intersex (and indeed some female) participants do not have a uterus, so asking about ‘sex’ in this case would also be ill-advised. Another example might be if you are studying breast cancer, where there is a multitude of factors that may determine someone’s risk. Trans women who were assigned male at birth and have undergone or are still undergoing gender-affirming hormone therapy (e.g., are taking estradiol) have an increased risk of developing breast cancer compared to cisgender men, who have not undergone this therapy [33]. Conversely, trans men are suggested to have an overall reduced risk of breast cancer, although this is strongly dependent on gender-affirming hormone therapy (e.g., testosterone), and whether the individual has had a mastectomy [34]. This example illustrates that in a study of breast cancer; multiple factors interact which cannot simply be captured by a question of either sex or gender. In the majority of cases, asking for a person’s sex will be unnecessary and muddle the collected data, and asking for a person’s (self-identified) gender should be the norm.

This rule is of particular importance for biological and psychological studies, where sex is often used as a binary category to approximate a number of different variables across genetic, hormonal, anatomical, and psychosocial axes [9,12]. We particularly caution against using sex in these cases, as it provides only a reductionist view, and may obscure phenomena that are not being measured, and simply being inferred.

### 3. Decide on the survey design and options

After you have decided on the relevant questions, it is important to consider how to design the survey. Free-text response options for all gender and sexuality related questions enable capturing a larger variety of responses, but may also lead to an increased number of invalid responses or reduced completion rate [7,13], as well as increased time investment for the analysis. A good alternative is a comprehensive multiple-choice question. If you are asking participants to pick their characteristics from a multiple-choice list, it is important to provide sufficient options. How many and what options to provide strongly depends on the type of survey and the population. Different people may have different preferences [35], with some people preferring longer lists to increase inclusion and visibility, whereas other people prefer shorter lists with a write-in option, to avoid unnecessary confusion. We provide two alternative versions (S1) for questions to ask regarding gender, sexuality and sex. In addition to these three questions, we also suggest adding two separate questions, one where individuals can indicate whether they are transgender; and another whether they are intersex, if this is relevant information.

Despite trying to give as many options as possible, it is usually impossible to capture the variety of people’s identities and experiences. Therefore, you should always give the options to not disclose and to self-describe with a free-text field. Even though this may make analysis harder in some cases, it will allow for a much more accurate description of the participants. Queer participants are also more likely to complete the survey and disclose their queer identity if they feel like they are able to accurately describe themselves [7]. Lastly, it is not uncommon for queer people to identify with multiple overlapping characteristics [17,36]. As such, it is good practice to allow for multiple options to be selected at the same time. This again captures the variety of experiences much better.

Many of the recommendations so far were written from a westernized viewpoint, and may not be appropriate depending on the group that is surveyed, or the language in which the survey is written. Many countries and cultures have their own identities which may not necessarily match with western ideas of gender or sexuality [24,37]. In international surveys it is important to keep this in mind, and accommodate for this through extensive multiple-choice options and a free-text field. In surveys of specific populations, it is advised to become familiar with local terms for gender and sexuality first, rather than using western or academic terms which may not align with people’s experiences. The same is true for language use. While some languages have straightforward gender neutral options, like English, Persian and Tagalog, others may not have such options available [38]. If you are writing a survey in such a language, national organizations or local interest groups may be able to advise on how to ensure inclusivity.

### 4. Provide information about how data is used, processed and stored

When surveying groups that may include queer individuals, it is important to provide information about how the data is used, stored and processed [25,30,39]. Queer people may be more easily identifiable based on their characteristics. It is for example not uncommon to be the only TGD person in a department or at a smaller conference. In addition, someone filling out the survey or questionnaire may be in a domestic situation or a country where coming out is dangerous or illegal. It is therefore important to have clear internal guidelines on how the information should be stored, processed, whether consent for storing or processing the data can be withdrawn, and how a request for withdrawal should be submitted and subsequently processed. These guidelines should be communicated to the participants. In the European Union such guidelines are also a legal requirement, per the General Data Protection Regulations [39]. For formal research programs, a detailed set of guidelines will likely be required by an ethics committee approving the research. Even for simpler situations, such as conference registration, it may be helpful to seek help from your local ethics committee to make sure you are complying with local regulations.

When the data is collected as part of a conference registration, it is advised to inform participants on if and how the entries are displayed to all participants, for example in a proceeding’s booklet or on the conference badge [40]. Queer people may use a name that is different from their legal name (in the case of TGD people the unused legal name is referred to as a deadname). If a participant’s name is required for legal purposes, it should be clearly stated, and an option for a displayed name (e.g., for the conference badge) should be provided in addition (see [40] for more guidelines on this).

### 5. Consider sex, gender and sexuality across all questions

Sex, gender, and sexuality may affect a large number of questions, so it is also important to consider these aspects in further questions. For example, when asking for relationship status, caregiver status, or family history, it is important to consider options that do not assume a heterosexual nuclear family (i.e., one mother, one father and child(ren)). Make sure to allow for options such as civil partnerships and polyamorous relationships. Do not assume all your participants had a mother and a father, or that mothers are primary caregivers. Having a write-in option for any question where sex, gender or sexuality may come into play is good practice. If your study concerns relationships with caregivers, you can ask participants to write in who the caregivers were (or are) in their family and subsequently reflect on that relationship when filling out other questions.

The same is true when asking about biological or anatomical characteristics. For example, do not assume that because someone indicates they are a man as their gender, that they are not able to get pregnant and do not have a menstrual cycle [41]. Generally, we would advise against having differential survey paths based on answers about gender or sexuality and instead pose the same questions with options to write in or not answer for each question. For example, if you are asking about pregnancy, include this question for everyone, rather than exclude the question based on the gender someone put in. This way you can avoid making unnecessary assumptions, and ultimately increase inclusion across the survey.

### 6. Determine how to deal with small sample sizes

It is generally assumed that queer individuals make up a minority of a given study population, resulting in a small sample size of queer, and especially TGD, individuals. This poses two main problems. First, as already mentioned in rule 4, one should be careful in reporting to not make anyone identifiable. This is especially important for (confidential) climate surveys among groups of peers, where individuals may be easily recognized by their gender identity or sexual orientation alone. That said, when reporting a summary of characteristics, it is important to not make an individual identifiable. Generally, it is recommended to suppress the precise number of individuals in any small group (i.e., fewer than 10 people), and instead simply state the number was smaller than 10 [42].

The second challenge is that groups may be too small for statistical analysis. Researchers might be tempted to try to fit participants in binary categories, or to combine categories. However, we strongly caution against this. Assigning participants to categories that do not fit with their self-identified gender (e.g., assigning all non-males to the female group) not only contaminates the data, but also violates the principle of Respect for Persons (as described in the Belmont Report [43] which summarizes the Ethical Principles and Guidelines for the Protection of Human Subjects of Research). Additionally, one should also avoid simply excluding small groups fully from analyses (effectively making being non-binary an exclusion criteria), as this would further exclude a group underrepresented in the scientific literature and violate the principle of Justice (as described in the Belmont Report). Lastly, we recommend against combining ‘other’ categories into one category post-hoc (e.g., combine all ‘other’ gender categories into one non-binary category). This would create a particularly heterogenous group, with a large variety of life histories and experiences [26]. In some cases, the best solution may be to discuss groups qualitatively, noting that insufficient numbers were available for statistical analysis. In other cases, it may be sufficient to note the small group size and that no conclusions could be drawn. If fully addressing the research question concerns groups for which collecting large samples is challenging (e.g., non-binary individuals), we encourage researchers to carefully consider how to increase their samples, preferably in collaboration with local queer communities.

If across studies, you repeatedly get a particularly small group size, or lack of individuals from certain groups, it is also good to reflect on why this may be the case. Are some groups unintentionally being excluded during participant recruitment or data collection?

### 7. Practice inclusive reporting on sex and gender

Reporting basic statistics on the sex or gender of research participants – whether human or non-human – is common practice and often required by publishers. Historically, this reporting has suffered from androcentrism, whereby men were considered the default [44]. More recently, there has been an emphasis on including women, but unfortunately this reporting is often still binary [28], even when the original collection was inclusive.

When reporting on data, even when samples only include binary participants, avoid using phrases that assume a sex or gender binary. For example, the phrase “data was collected from 50 participants (30 women)” implies that the other 20 participants were men. Instead, one should specify all groups that were included, for example “data was collected from 50 participants (30 women, 18 men, 2 non-binary people)” [28]. If no participants were found for certain gender categories, it is better to be explicit (e.g., ‘50 participants (30 women, 20 men, no non-binary people)’), or include a demographic table.

It is also preferred to avoid language which implies a binary split of sex or gender (e.g., phrases like ‘both sexes’ or ‘opposite sex’). This is both the case when reporting on sex and gender, and when describing queer relationships (e.g., avoid terms such as ‘same-sex attraction’ in human contexts). Lastly, we recommended avoiding gendered language for biological terms (e.g., use androgens instead of male hormones). This will also encourage being more deliberate in what is actually being reported on, and avoids unnecessarily conflating terms.

### 8. Account for fluidity of gender in longitudinal studies

As mentioned before, what a person reports as their sexual orientation, gender, and in some cases sex, can change over a person’s lifetime, either due to a change in their self-identification, due to a person’s eventual self-realization, or due to a person coming out and publicly sharing these aspects of their identity. It may also be the case that a person’s gender changes either day to day, or over longer periods of time [45]. When measuring participants over a longer study, it is therefore important to account for these changes.

When conducting a longitudinal study involving human participants, we recommend asking for their demographics at every session, if this is relevant for the study, and avoiding assuming their sex, gender, and sexual orientation based on previous answers. In addition, be aware that consequently, a participant may also change their name and/or pronouns. This is true for all longitudinal studies, but especially those involving children or adolescents through their development [46]. If data is analyzed based on gender for each collection, we advise to include participants in the group with which they identified at the time of collection, to account for gender fluidity. In some cases, it may be more appropriate to retroactively adjust the gender, but this will depend on the research question, and should only be done with consent from the participant.

It is also recommended to consider these issues when preparing data collection for single events, such as conferences. In most cases, registration will take place several months ahead of the conference, and it is possible participants may change their name, pronouns, or the gender with which they identify in that time. To accommodate for this, make sure to have clear information for participants on how they can change this information, and have a clear and fast process for doing so.

### 9. Balance the demands from reviewers, journals, and/or funding agencies

Often both funding agencies and journals have guidelines or requirements regarding reporting sex and gender. Some funding agencies, including the US National Institutes of Health (NIH), assume a sex/gender binary in their reporting requirements [47]. Others, such as the European Research Council, champion the inclusion of non-binary genders in data collection, analysis and reporting [48]. Unfortunately, depending on the journal or funding agency, the required terminology may not be in line with the guidelines we have laid out in this paper.

In cases where a binary approach is required, avoid misgendering participants, for example by including them into one of these groups, and othering participants [28], for example by including them as only as ‘other’. We recommend that researchers avoid compromising their participants’ self-identification in their reporting. Instead, pointing these issues out to reviewers, journals, and funding sources may prompt them to change their perspectives and policies.

Balancing these demands is increasingly important in hostile environments, for example as the National Institutes of Health in the US are cancelling grants which cover sex, gender, diversity and sexuality [49]. Especially in these conditions, it is crucial to continue to study these variables in an inclusive way and continue to point out these issues.

### 10. Include sex and sexuality in studies on animals

Many of the tips given so far are aimed at research on humans, however, it is important to consider sex and sexuality in animal research too. First, it is important to note that gender is a concept that only applies to humans, so for animal research sex is the relevant variable. Over the last decade or so, there have been increasing calls for including males and females in research, to address a pervasive sex bias [50,51]. In the US, this has led to the NIH Notice on Sex as a Biological Variable (SABV), which required all proposals including human or vertebrates from 2016 to include sex as a variable [52,53]. This has led to many new exciting discoveries on sex differences [54], such as the characterization of the effects of sex on 234 traits in over 50,000 mice [55]. In the field of ecology, a focus on sex determination in turtles revealed the devastating effects of climate change [56].Unfortunately, the majority of studies still lack meaningful sex-based analysis [50].

When sex-based analysis is applied, sex is often assigned based on the external appearance of the genitalia, or if available, chromosome testing, which leads to a simplification of the complex and diverse biological processes which are involved in sex determination. New multi-layer frameworks have been proposed to more accurately capture these processes and give a richer understanding of their biology [11,12,57,58]. The models include considerations of not just the animal’s genitalia or gametes, but also their behavior, hormones and genetics. Such an approach has for example been applied to the study of type 2 diabetes (T2D), revealing the differential effects of testosterone in male and female mice [59].

A multimodal framework of sex, behavior and hormones is also necessary for the study of species that do not follow what we would think of as conventional sex binaries [60]. For example, the female spotted hyena (*Crocuta crocuta*) is generally more aggressive and dominant, and possesses an external phallus-like clitoris, and no vaginal opening [61]. Conversely, the striped hyena (*Hyaena hyaena*) undergoes a phase of convergence in adolescence, where the genitalia of males and females start to resemble one another, before diverging again in adulthood [62]. Thus, any sex-based comparison of these species requires a multimodal approach.

Although there is no single solution to approaching sex in animal research, we encourage animal researchers to engage with such frameworks and incorporate them into their work in a meaningful way. This will not just increase overall inclusivity, but may also make the work more interpretable and generalizable [63].

Sexuality is understudied in many animal species, and as such is seen as rare [64,65]. As in humans, this is part of a vicious cycle, where these behaviors are not studied, because they are assumed to be absent or rare, leading to a paucity of observations confirming this idea [64]. Noting such behaviors and including them in analyses is important, as it will increase our understanding of an animal’s behavior, ecology and evolution [66,67].

## Concluding remarks

We have set out guidelines to promote queer inclusivity in data collection, processing, analysis and reporting within STEM. Our aim with these guidelines is to inform STEM researchers who do not specialize in queer topics on how to best perform inclusive data analysis in their field of research. It should be stressed again that these are neither the only guidelines (see [7,13,17,10,25–30] for more helpful guidelines), nor are they hard rules, as demands may vary depending on the population, social climate, and context of the survey.

In addition, an increasing number of papers, such as this one, is being published in STEM-focused journals, discussing how academics can improve their support and inclusivity [6,16], for example while organizing conferences [40], or during faculty hires [68]. These are a great resource to consult when in doubt, or to refer back to occasionally as a refresher.

Language and societal norms around sex, gender and sexual orientation are ever changing [6]. Many institutes house (gender) equality officers or committees, who may be able to advise. In addition, many professional societies have equality committees who will likely be up to date on these issues and how they pertain to your particular field. We advise readers to use these guidelines as a starting point, and to continue the conversation with participants, fellow researchers and queer interest groups.

## Supporting information

S1 File ALBA Guidelines: Designing inclusive forms for gender and sexual diversity. Guidelines on how to designing surveys and forms with LGBTQIA+ people in mind.
(PDF)

S2 File ALBA Guidelines: Reporting on sex and gender in neuroscience research. Guidelines describing how to report on sex and gender across various aspects of neuroscience research.
(PDF)

## Abbreviations

DSD: difference in sex development
LGBTQIA+: lesbian, gay, bisexual, transgender, queer, intersex, asexual
NIH: national institutes of health
SABV: sex as a biological variable
STEM: science, technology, engineering, and math
T2D: type 2 diabetes
TGD: transgender and gender diverse.
