# Supplementary material for: Ten simple rules for queer data collection and analysis by STEM researchers
Source: PLoS Comput Biol. 2025 May 28;21(5):e1013091. doi: 10.1371/journal.pcbi.1013091 (PMC12118915; doi:10.1371/journal.pcbi.1013091)
Supplement: S1 File — Guidelines on how to designing surveys and forms with LGBTQIA+ people in mind. (PDF) [file pcbi.1013091.s001.pdf]

ALBA Guidelines: Designing inclusive forms for gender and sexual diversity

(I) Why is this important & what type of forms would this guideline be applicable to?

It has become more common to gather demographic information in a variety of settings related to neuroscience, such as conference registrations and job application forms. In addition, many neuroscientists work with human participants and need to gather accurate demographic information on them. Collecting such information is an important part of improving diversity and inclusion; after all, if one does not know who is or is not included in certain settings, it is hard to address underrepresentation or disadvantage with targeted affirmative actions.

With this guideline, we aim to set out best practices for improving the inclusion of LGBTQIA+ (Lesbian, Gay, Bisexual, Trans, Queer/Questioning, Intersex, Asexual and other identities) people, that is, individuals with varying genders, sexualities, and transgender and intersex people. Please note, that it is a work in progress; practices and identities may change over time, depending on region and culture, and as such, feedback is very welcome. The guidelines are applicable to any setting in which such identities are surveyed, and we encourage its use for conference registration forms, HR surveys and demographic surveys for human participants in research projects. The goal is to help anyone unfamiliar with these identities improve their forms or surveys to more accurately capture these individuals.

(II) Best practices

1. General recommendations

Provide information about how data is used, processed and stored.
LGBTQ+ individuals, especially those with intersectional identities, may be easily identifiable from surveys that are supposed to be anonymized. Giving information about how the data is handled can help mitigate worries about this.

Consider what is relevant to your survey.
Consider what the goal of the survey is, and what questions are relevant. Generally, LGBTQ+ surveys are used for gauging diversity of e.g. research participants or conference attendees, and as such questions about sexuality, gender, and transgender status may be relevant. However, in a non-medical setting, questions about birth sex are often not relevant. If the survey is used to determine effects of either sex or gender on something, consider what the most relevant factor is. For example, sex does not map directly onto hormones, so if you're performing an experiment to determine the effect of testosterone on a dependent variable, asking birth sex is not accurate enough to answer your question.

Consider sex, gender and sexuality across all questions.
When performing a survey, sex, gender, and sexuality may come into play in questions in unexpected ways. For example, when asking about relationship status, it is important to consider that not all relationships are between two cisgender, heterosexual partners, and that arrangements such as civil partnerships, should be included in the options. Similarly, questions should avoid assumptions on the structure of participants' families (e.g. some families have two parents of the same gender; families can include caregivers who are not parents or even biologically related). Make sure that you check all questions for potential biases and exclusionary or binary language.

Always give the options to self-describe and to not answer.
As much as we try to include every option, it is impossible to capture the full diversity of people, so adding an option to self-describe their identity will allow everyone to be included, and will allow your data to be more accurate. In addition, acquired data can be used to improve further surveys. Having just a single fill-in-the-blank option to self-describe, especially for gender options beyond the binary, rather than a list of possible options is often preferred, as it allows for more accurate data, even though this can make analysing the data more laborious. This option should preferably be indicated as "additional category/identity not listed", rather than "other", to avoid — quite literally — other people outside of the listed categories.

Allow for multiple options to be selected.
Often people do not fall neatly within a single box, but rather have an identity that can be described by multiple terms. Allowing people to tick more than one option allows you to capture this — for example, someone may identify with a binary gender option, as well as with the descriptor trans*.

Consider what options to include.
In our recommendations below, we give fairly extensive lists of options for gender and sexuality. However, different schools of thoughts exist on this topic. Including more terms signals inclusion of a wide range of identities, however, it can quickly become confusing and make filling in the survey harder. As such, you might consider a shorter list, in which case it is important to consider what demographics you are expecting, and how are you going to make sure to include all options both in the survey (a free text option), and in your subsequent analysis. In this case, it is also advisable to include a disclaimer, for example: *"We are aware that gender identity and sexual orientation encompass many terms and nuances, all of which we consider equally valid. We have selected a set of options that should be interpreted in broad terms, and that are meant to facilitate statistical analysis of LGBTQIA+ realities. Nevertheless, if you consider that the suggested options don't adequately represent your own experience, you are very welcome to self-identify in the corresponding field"* (PRISMA, https://prismaciencia.org/).

Forms in other languages
The recommendations given are predominantly for English language forms, and include many terms which may not directly translate into other languages. Conversely, many other languages will have their own terms for certain LGBTQ+ identities. As such, it is important to engage with local LGBTQ+ interest groups to determine how best to survey LGBTQ+ people in that particular language.
In addition, English is a largely non-gendered language, which is not the case for many languages across the world. To promote inclusivity, forms and questionnaires should ideally be written in a gender-neutral way. If that is not possible, multiple versions of forms and questionnaires should be made available based on personal gender pronouns. In some cases, LGBTQ+ people have come up with their own way to denote gender neutrality, in other cases, extra care should be taken to be as inclusive as the language permits. National or local LGBTQ+ groups may have guidelines on the best terms to use in their particular language, and it is advisable to seek this out before creating the survey.

2. Specific recommendations

Gender
Gender identity describes an individual's personal experience of their gender and is separate from their sex assigned at birth, gender expression, and their physical appearance. Although these often line up (when a person's sex assigned at birth and gender line up, they are cisgender), this is not always the case. People who do not have the same gender as their sex assigned at birth are transgender. However, 'transgender' itself is not a gender, but rather an adjective (someone may be a transgender woman for example). Therefore, one should not include 'transgender' in the gender options. It is recommended to include a question asking whether someone is transgender as a separate question, if this is something that is relevant to your survey. In addition, 'male' and 'female' are used for sex, but it is preferred to use 'man' or 'woman' for the gender options.

Below are examples of how to survey gender identity. Note these are not exhaustive lists and contain mainly North American/English identities. If you are surveying an international audience, or a specific country or region, educating yourself on what identities exist within your surveyed areas is advised. Some examples of other genders include: "hijra, thirunar/thirunangai/thirunambi, kinnar, khwaja-sira, nupa manba/nupi manbi, jogappa, mangalamukhi, and aravani in South Asia; muxe in southern Mexico; fa'afafine in Samoa and American Samoa; bakla in the Philippines; waria, bissu, calabai, and calalai in Indonesia; femminielli in Naples; and sworn virgins in the Balkan states." (pg 1328) [1]

Long version	Shortened version
Gender identity (select all that apply)<br>• Agender<br>• Androgynous<br>• Bigender<br>• Genderfluid<br>• Genderqueer<br>• Gender non-conforming<br>• Man<br>• Non-binary<br>• Pangender	Gender identity (select all that apply)<br>• Agender<br>• Genderqueer<br>• Man<br>• Non-binary<br>• Woman<br>• Two-spirit<br>• Do not know/questioning<br>• Prefer not to say<br>• Additional gender category/identity not listed

• Two-spirit<br>• Woman<br>• Do not know/questioning<br>• Prefer not to say<br>• Additional gender category/identity not listed (please specify below)	(please specify below)

Do you consider yourself to be transgender? (single-choice)

- Yes
- No
- Do not know/questioning
- Prefer not to say

Sexuality

Sexuality or sexual orientation describes the gender(s) that a person is generally attracted to. Sexuality includes a wide spectrum, and distinctions can be made between romantic and sexual attraction (e.g. a biromantic, asexual person describes a person who is romantically attracted to people across two or more genders, but not sexually attracted to anyone). It can therefore be hard to capture the full spectrum in a multiple-choice list, but including an option to self-describe and allowing people to choose multiple options can mitigate this. Below is an example of how to survey sexuality

Long version	Shortened version
Sexual Identity/Sexual Orientation (select all that apply)<br>• Asexual<br>• Bisexual<br>• Demisexual<br>• Fluid<br>• Gay<br>• Greysexual<br>• Lesbian<br>• Pansexual<br>• Queer<br>• Questioning or unsure<br>• Straight/heterosexual<br>• Prefer not to say<br>• Additional category/identity not listed (please specify below)	Sexual Identity/Sexual Orientation (select all that apply)<br>• Asexual<br>• Bisexual<br>• Gay<br>• Lesbian<br>• Pansexual<br>• Queer<br>• Straight/heterosexual<br>• Prefer not to say<br>• Additional category/identity not listed (please specify below)

Sex

If sex assigned at birth is relevant (e.g. for medical reasons), this can be surveyed. You need to carefully consider what question to ask, as the wording may change the answer a person gives (e.g. a person's sex assigned at birth may be different from their legal sex). Oftentimes we use sex as a shorthand for other relevant information. For example, you might be researching something related to uterine health and are considering using legal sex as an inclusion criterion. However, using legal sex to select your population would exclude trans men who have had their legal sex changed, and using either definition of sex (legal sex or sex assigned at birth) would include women who have had hysterectomies, thus not accurately capturing the population of people with uteruses.

Sex assigned at birth:

- Male
- Female
- Intersex

- Not listed
- Prefer not to say

Intersex people
Some people were born with physical differences in sex anatomy, reproductive organs, chromosomes and/or hormone function, which do not fit typical expectations. These people are called intersex or having Difference in Sex Development (DSD). Often they are included under the LGBTQIA+ umbrella, but they are not captured by either gender, sexuality or sex assigned at birth questions. Based on guidelines by InterACT, an advocacy group for intersex youth, we recommend including a separate question to accurately measure this group. It is also recommended to include a (short) description, because some people, even those who are intersex, may not be familiar with the term.
Were you born with a variation in your physical sex characteristics? (This is sometimes called being intersex or having a Difference in Sex Development (DSD).)

- Yes
- No
- I don't know
- Prefer not to say

Name
On non-anonymized surveys or forms, such as conference registration forms, you may need to ask for a person's name and/or pronouns. Consider that a person's legal name may not correspond to their preferred name, and always allow a person to fill in their preferred name if possible. If a legal name is required (for example for official documentation), make sure to include information on how the name is used.

Pronouns
In some cases, a form may need to ask for pronouns, for example for a conference registration where pronouns are printed on the badge. A person's pronouns and gender identity may not always align, so avoid inferring pronouns. Clearly state what the pronouns may be used for (e.g. will they be listed, used by moderators during introductions, printed on labels), as some people may be less comfortable disclosing their pronouns when they are subsequently publicly available. Pronouns should ideally be surveyed using an optional free text field.

(III) Resources

NASEM consensus study report 'Measuring Sex, Gender Identity, and Sexual Orientation'. Includes data and recommendations on how best to survey sex, gender identity and sexuality.
https://nap.nationalacademies.org/catalog/26424/measuring-sex-gender-identity-and-sexual-orientation

Further reading on creating an LGBTQIA+ inclusive survey by Vanderbilt university
https://www.vanderbilt.edu/lgbtqi/resources/how-to-ask-about-sexuality-gender

Article on various gender identities in Asia
https://kontinentalist.com/stories/transgender-people-in-asia

More information on intersex people
https://interactadvocates.org/faq/

How to build an inclusive conference
https://journals.asm.org/doi/10.1128/msystems.00433-23

A quick guide to data collection for LGBTQ+ characteristics
https://figshare.com/articles/journal_contribution/A_quick_guide_to_data_collection_for_LGBTQ_characteristics/24633756

(IV) Glossary of terms

Overview of LGBTQIA+ terms and their definitions
https://health.ucdavis.edu/diversity-inclusion/LGBTQI/LGBTQ-Plus.html

Overview of trans-specific terms and their definitions, and recommendations for language to avoid
https://www.glaad.org/reference/trans-terms

Overview of gender identity terms across the globe
https://www.digitaltransgenderarchive.net/learn/terms

References

1. Aghi K, Anderson BM, Castellano BM, Cunningham A, Delano M, Dickinson ES, et al. Rigorous science demands support of transgender scientists. Cell. 2024;187: 1327–1334.
