# Supplementary material for: Ten simple rules for queer data collection and analysis by STEM researchers
Source: PLoS Comput Biol. 2025 May 28;21(5):e1013091. doi: 10.1371/journal.pcbi.1013091 (PMC12118915; doi:10.1371/journal.pcbi.1013091)
Supplement: S2 File — Guidelines describing how to report on sex and gender across various aspects of neuroscience research. (PDF) [file pcbi.1013091.s002.pdf]

# ALBA Guidelines: Reporting on sex and gender in neuroscience research

Sex is often used as a binary covariate across neuroscience, psychology and psychiatry research, in studies both involving humans and animals. However, considering sex as a binary of male and female is a reductionist view that does not accurately represent the complex realities of sex and gender, and how they may influence the brain. Here, we provide guidelines and resources to encourage neuroscientists to reconsider how they analyse and report on sex and gender in their research. These guidelines follow from our previously published guidelines on how to collect accurate data on sex and gender (https://www.alba.network/GSDinclusiveforms). With both guidelines, we aim to set out best practices for improving inclusion and diversity in research. They are by no means exhaustive but serve at a starting point in a continuous effort to make science more diverse and equitable.

## What is gender?

Gender identity describes an individual's personal experience of their gender and is separate from their sex assigned at birth, gender expression (how they present themselves), and their physical appearance. Although these characteristics often line up (when a person's sex assigned at birth and gender line up, they are cisgender), this is not always the case. Neither sex nor gender is binary, and it is important to consider the natural variation in both. For more detailed information on gender identity, sex, and how to collect data on these, please review our previous guidelines: https://www.alba.network/GSDinclusiveforms.

## Consider the question

While collecting and analysing data on the sex or gender of experiment participants, it is crucial to keep in mind what the ultimate question is that the analysis is aiming to answer. For example, if you are looking to determine the experiences of people with uteruses, it is inaccurate to use the gender of a participant as a proxy. This is particularly important to keep in mind when analysing the societal impact on people of different genders, as this may depend on how they are socialised in early life (e.g., trans people who transitioned later), or how they are viewed by society at large (e.g., someone who is non-binary, but is often assumed to be a woman).

In general, when studying human participants, using their self-reported gender is the best practice. If it is important to know the sex assigned at birth, it may be useful to give an explanation to the participant of why this is collected, so that the participant can give the information that is most pertinent to the experiment.

## Language around sex and gender

Reporting basic statistics on the sex or gender of research participants – whether human or nonhuman – is common practice and often required by publishers. When reporting these data, try to avoid phrases that assume a sex/gender binary (e.g., that people who are not women identify as men). Instead of reporting that "data was collected from 50 participants (30 women)", one could report that "data was collected from 50 participants (30 women, 28 men, two non-binary people)". The same applies when reporting participants' sex (approximately 1% of the population are intersex). In addition, it is better to avoid gendered language for biological terms (e.g. use androgens instead of male hormones), and language which implies a binary split of sex (e.g. phrases like 'both sexes' or 'opposite sex').

## Dealing with small sample sizes

Another challenge can occur in conducting gender- and sex-based statistical analyses. Many studies explore the effects of sex or gender on their experimental outcomes. Though statistical analyses are often sufficiently powered for binary male vs. female analyses, this is often not the case for non-binary or intersex participants. Statistical analyses for these groups may be underpowered and/or risk participant confidentiality. We therefore recommend conducting sex/gender-category-specific statistical analyses only for samples of at least 10 individuals. For smaller samples, the rationale for not conducting statistical analyses should be reported. Most studies will have a small minority of non-binary or intersex participants. Authors should be cautious when sharing demographic details (e.g., age, race, ethnicity) related to these participants, as those may compromise their anonymity in some cases.

Two common alternatives to avoid dealing with small samples of sexual or gender minorities should be avoided altogether. Researchers should never assign participants to one of the two predominant gender/sex categories unless it is in line with their self-identified gender or sex, as it would violate the principle of Respect for Persons (as described in the Belmont Report [1] which summarizes the Ethical Principles and Guidelines for the Protection of Human Subjects of

Research). Additionally, research should avoid recruiting a sample intentionally consisting only of binary participants, essentially excluding non-binary or intersex participants (e.g. making non-binary identity an exclusion criterion). This would further exclude a group underrepresented in the scientific literature and violate the principle of Justice (as described in the Belmont Report [1]). Data for sexual minorities should be collected and reported in aggregate along with other participants and only excluded for sex/gender-category-specific statistical analyses, as detailed above.

## Sex and gender in longitudinal studies

Some studies, especially in humans, require data collection over multiple sessions spanning many years. Participants should be asked about their self-identified gender in each session, if this is relevant for the study. For example, researchers should not assume that a participant recruited as a man would still self-identify as one year later. This is true for all longitudinal studies, but especially those involving children or adolescents through their development. If data is analysed based on gender, it is best to include participants in the group with which they identify at the time of collection.

## Sex and gender in animal research

In animal research animals are often simply divided into two classes, male and female, usually based on their external sexual characteristics (e.g. genitalia). However, like in humans, sex is actually a multidimensional phenomenon, with wide variations being observed across genetic, endocrinological, physiological and social levels. For example, although genetically female, some lionesses have been observed to grow manes and take on a more masculine social role [2]. This is just one example where sex is not as clearly defined (for more examples, see the book ‘Evolution’s Rainbow’ by Joan Roughgarden), and depending on what is being studied, one cannot simply divide these animals into male and female.

Because sex is diverse, even in many standard lab animals, it is advisable to take a multivariate approach, especially when studying the brain in the context of sex. A number of variables which are implicitly assumed when dividing by sex, may be measured to gain a more nuanced picture, for example hormone levels, reproductive status, and social status.

In addition, one may encounter non-binary expressions of sex in lab animals, such as hermaphrodite animals. In most cases these are simply excluded from studies, but by including them and measuring sex as a multivariate in these animals, they provide invaluable insight into the effect of different aspects of sex on the studied variables.
How to measure and treat sex in animal research may depend on the animal model, the studied system and a number of other factors. However, a number of multivariate frameworks have been proposed that may guide you in designing a study with a more nuanced approach to sex [3,4].

## Balancing demands from reviewers, journals, and/or funding agencies

Some funding agencies, including the US National Institute of Health (NIH), assume a sex/gender binary in their reporting requirements (see this example). For human participants, NIH allows three options when reporting gender: Male, Female, and Unknown. This terminology is not in line with that used to describe gender and doesn’t include non-binary options. Hopefully, this terminology will be changed with time, but as it stands, we recommend including any participant who does not identify as a man or a woman as “Unknown” rather than misgender them. More broadly, we recommend that researchers avoid compromising their participants’ self-identification in their reporting. Instead, pointing these issues out to reviewers, journals, and funding sources may prompt them to change their perspectives and policies.

## References


1. The National Commission for the Protection of Human Subjects of Biomedical and Behavioral Research. The Belmont Report. US; 1979. Available: https://www.hhs.gov/ohrp/sites/default/files/the-belmont-report-508c_FINAL.pdf
2. Gilfillan, Geoffrey D., et al. "Rare observation of the existence and masculine behaviour of maned lionesses in the Okavango Delta, Botswana." *African Journal of Ecology.* 2017; 55.3
3. Smiley KO, Munley KM, Aghi K, Lipshutz SE, Patton TM, Pradhan DS, et al. Sex diversity in the 21st century: Concepts, frameworks, and approaches for the future of neuroendocrinology. *Hormones and Behavior*. 2024;157: 105445. doi:10.1016/j.yhbeh.2023.105445
4. McLaughlin JF, Brock KM, Gates I, Pethkar A, Piattoni M, Rossi A, et al. Multivariate Models of Animal Sex: Breaking Binaries Leads to a Better Understanding of Ecology and Evolution. *Integrative and Comparative Biology*. 2023;63: 891–906. doi:10.1093/icb/icad027
